# Four-Dimensional Flow in Fontan Patients: Advanced Haemodynamic Assessment

**DOI:** 10.3390/jcm14113801

**Published:** 2025-05-29

**Authors:** Dominik Daniel Gabbert, Anselm Sebastian Uebing, Inga Voges

**Affiliations:** 1 Department of Congenital Heart Disease and Pediatric Cardiology, University Hospital Schleswig-Holstein, 24105 Kiel, Germany; dominik.gabbert@uksh.de (D.D.G.); anselm.uebing@uksh.de (A.S.U.); 2 DZHK (German Centre for Cardiovascular Research), Partner Site Hamburg/Greifswald/Kiel/Lübeck, 24105 Kiel, Germany

**Keywords:** Fontan circulation, single ventricle, cardiovascular magnetic resonance, 4D Flow

## Abstract

Staged palliation with the creation of a Fontan circulation is the standard surgical approach in patients with a single ventricle. The Fontan circulation is a complex circuit that is associated with various complications that may present early or later in life and can limit life quality and expectancy. In this context, a good understanding of the Fontan physiology is important to improve outcomes for single-ventricle patients. Cardiovascular magnetic resonance (CMR) is recommended for the long-term follow-up of Fontan patients, as it provides functional and haemodynamic information. Four-dimensional (4D) Flow MRI is a time-resolved, three-dimensional, velocity-encoded cardiovascular magnetic resonance technique that is increasingly used in Fontan patients because it not only enables measuring blood flow within a three-dimensional (3D) volume, but also allows for assessing more advanced haemodynamic parameters that may help in understanding the Fontan physiology and pathophysiology. Furthermore, 4D Flow is used for image-based simulations using computational fluid dynamics. In this review, we provide an overview of the use of cardiovascular magnetic resonance flow assessment, with a focus on four-dimensional flow (‘4D Flow’).

## 1. Introduction

Univentricular heart disease is a group of conditions that result in a functionally single ventricle [1]. The most common disease within this group is hypoplastic left heart syndrome. Patients are usually treated using a staged approach involving several sequential palliative operations, the aim of which is to achieve the so-called Fontan circulation [2,3]. The Fontan circulation, characterised by the rerouting of venous blood directly into the pulmonary circulation without the assistance of a sub-pulmonary ventricle, presents a unique set of haemodynamic challenges, characterised by non-pulsatile, low-shear pulmonary blood flow; systemic venous hypertension; and low cardiac output [4]. The resulting low-pressure system in the pulmonary arteries can lead to a variety of complications such as venous congestion, altered flow patterns, and an increased risk of liver fibrosis and pulmonary hypertension [2,4,5]. The haemodynamic intricacies of the Fontan circulation highlight the importance of advanced imaging techniques.

Cardiovascular magnetic resonance (CMR) imaging is a key imaging modality in patients with congenital heart disease and part of the follow-up program for patients with a single ventricle who have the Fontan circulation [2,6,7]. Besides anatomic and functional CMR assessments, CMR flow measurements are essential for haemodynamic evaluations in Fontan patients. Two-dimensional (2D) phase-contrast mapping is still routinely used to measure blood flow. However, four-dimensional (4D) flow phase-contrast measurements (4D Flow) are becoming increasingly popular, and several studies have shown that, in comparison to 2D phase-contrast mapping, 4D Flow can accurately measure standard CMR blood flow variables [8,9,10]. In general, 4D Flow is an extension of 2D phase-contrast mapping and allows for a more comprehensive assessment of blood flow, which, besides standard blood flow variables (forward flow, backward flow, and net flow), includes the visualisation of blood flow velocities by colour coding and blood flow patterns, as well as the measurement of advanced haemodynamic markers such as wall shear stress, kinetic energy, energy loss, and others [11,12,13]. These parameters can offer insights that go beyond basic flow measurements, contributing to a deeper understanding of the Fontan physiology. This review article provides a topical overview of the clinical and scientific use of 4D Flow in Fontan patients, together with an outlook.

For this review, a literature search on PubMed covering the period from 2010 to August 2025 was performed. The inclusion criteria were as follows: (1) original articles, (2) reviews, (3) expert consensus articles, and (4) articles in English.

## 2. Acquisition and Applications of 4D Flow

### 2.1. Acquisition and Reconstruction

Fontan palliation results in a haemodynamically complex circulation which, in principle, encourages the use of 4D Flow [14]. Multiple regions of interest can be covered by a single acquisition slab. However, conditions in arteries, veins, or ventricles, as well as post-processing requirements, may vary greatly depending on the analysis and may favour the use of different acquisition parameters, in particular concerning velocity encoding, as well as temporal and spatial resolution [11,12].

By utilising multi-VENC (velocity encoding) techniques, it is possible to acquire data that capture both high-velocity flows in the aorta and slower flows in the total cavopulmonary connection (TCPC), thus improving the overall quality of haemodynamic data in a single acquisition [15,16].

It should be noted that, since the pulsatility of blood flow in the TCPC is more dependent on respiration than on the heartbeat, blood flow amplitudes and peak velocities will be underestimated in ECG-gated measurements [17].

As the scan protocols of Fontan patients are relatively comprehensive, short scan durations of up to 10 min are desirable for the use of 4D Flow in clinical routine in order to minimise motion artefacts and improve patient compliance [11,14]. For use in prospective research studies, scan duration is less important while the comprehensiveness of data is prioritised [11]. Acquisition parameters should be chosen carefully, matching such priorities. Furthermore, acquisition parameters should take post-processing requirements into account. The requirements of common or advanced analysis quantities on acquisition parameters are discussed in detail in the 4D Flow cardiovascular magnetic resonance consensus statements [11,12].

Reconstruction typically covers three reconstructions, with phase-encoding along the three spatial directions and a magnitude reconstruction to present the anatomy [18]. The determination of turbulence kinetic energy places further demands on the reconstruction. In total, the following four magnitude reconstructions are required: one with velocity compensation and three magnitude reconstructions related to the three phase-encoding steps with gradients along the three spatial directions [19,20].

When applying 4D Flow in clinical or research settings, careful planning of the acquisition strategy is crucial. Factors such as patient cooperation, the use of respiratory gating, and ECG synchronisation should also be considered, as they may significantly impact image quality and data reliability. Furthermore, increasing attention is being paid to the trade-offs between spatial and temporal resolution, which must be optimised based on the clinical or investigational question at hand. In smaller patients, 3D Flow may be a preferable alternative to 4D Flow, as it is faster and provides an improved image quality. A recent study showed that there was a good-to-excellent agreement between 4D Flow and 3D Flow for flow measurements [21].

### 2.2. Post-Processing, Clinical, and Advanced Parameters

The post-processing of the clinical workflow covers corrections of velocity data, the segmentation of volume or the cross-sectional area, and the determination of parameters. Corrections include background phase offset correction and velocity anti-aliasing. Phase offset errors occur due to eddy currents and concomitant gradient fields and should be corrected for by linear or polynomial fits to static tissue [22].

Velocities that exceed the VENC parameter will experience phase shifts greater than 180° and cannot be mapped unambiguously. Phase-unwrapping makes use of the fact that adjacent voxels are expected to show small changes in velocities. Large differences are assumed to be due to exceeding the VENC velocity and are corrected accordingly [23].

**Figure 1:**
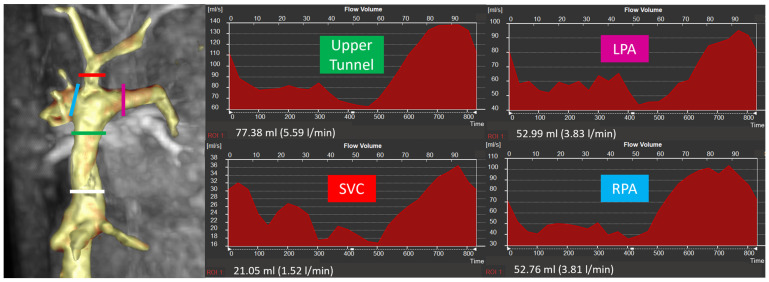
Site of flow measurements in the TCPC and example of flow curves (white line = lower tunnel, green line = upper tunnel, red line = superior vena cava, pink line = left pulmonary artery, blue line = right pulmonary artery).

**Figure 2:**
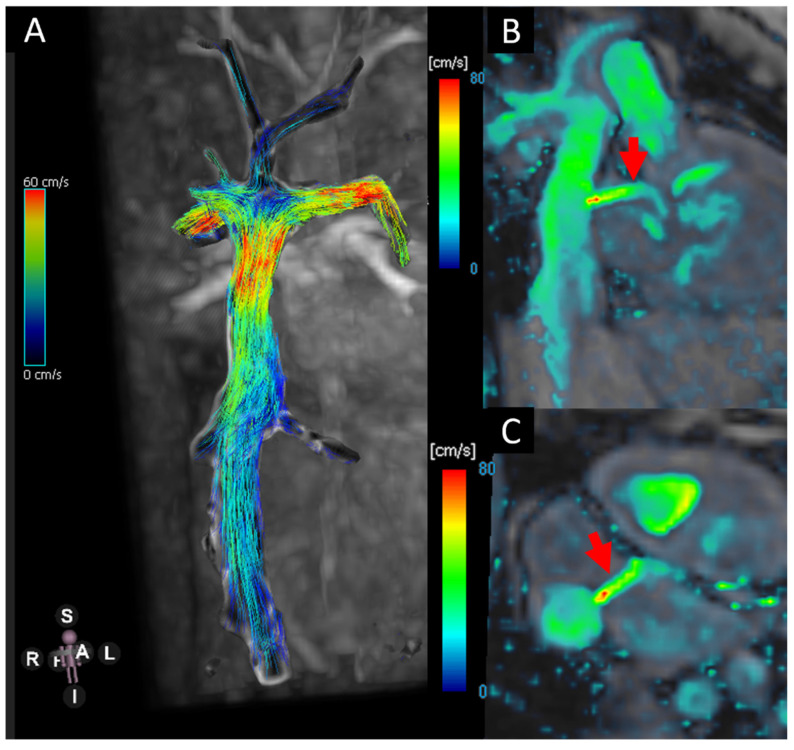
(**A**) Blood flow visualisation using stream lines in the TCP.C. (**B**,**C**) Velocity heat map showing the flow across the fenestration (red arrows).

Flow volume in the cardiac cycle and peak velocities are the most important clinical parameters in the assessment of the Fontan circulation. Furthermore, the visualisation of blood flow patterns can be helpful in clinical assessments (Figure 1 and Figure 2) [14]. This includes the visual detection of abnormal vascular anatomies or shunts. Four-dimensional Flow particle tracing is a method that can help visualise blood flow and provide quantitative measures. Roos et al. provide an overview of this method [24]. Several other fluid dynamics quantities have been used in research. Quantities that are calculated on a voxel basis are kinetic energy and turbulence kinetic energy [19,20,25]. Quantities that depend on the spatial arrangement of the velocity field map and involve velocity in adjacent voxels are vorticity, helicity, circulation, viscous energy loss, wall shear stress, and relative pressure [26,27,28,29,30]. For the systematic analysis of flow dynamics in large vessels, a method has been proposed in which flow variables are determined continuously along the course of the vessel by projection onto cross-sectional planes [26].

### 2.3. Modelling

Computational fluid dynamics (CFD) simulations, when combined with 4D Flow MRI data, offer a powerful tool for investigating the haemodynamics of the Fontan circulation. By using 4D Flow-derived velocity and pressure data as boundary conditions, CFD models can simulate the fluid dynamics within the Fontan circuit, offering insights into energy loss, shear stress, and pressure distributions. This allows for a more individualised approach to predicting patient outcomes, including potential complications such as liver fibrosis, pulmonary hypertension, and reduced exercise tolerance. Patient-specific computer models have been used to predict the outcomes of surgeries or interventions [31]. Blood flow measurements, typically at vascular cross-sections of inlets and outlets from CMR in terms 4D flow or 2D phase-contrast acquisitions, as well as segmentation, can serve as boundary conditions for the development and validation of models [31,32,33].

The specific advantage of using a single 4D Flow measurement is the simultaneous measurement of blood flow at different positions. Physiological changes and changes in patient position during the MRI examination may lead to inconsistencies between 2D phase-contrast measurements, resulting in non-physiological simulated haemodynamics, although, to the best of our knowledge, this has not been discussed in the literature. If the simulation involves the TCPC and no respiratory gating for resolving the full respiratory cycle is available, the use of real-time 2D phase-contrast measurements may be considered [17]. For segmentation, the use of phase-contrast magnetic resonance angiography (PC-MRA) may be convenient when the acquisition included a 4D flow sequence [32,34]. The disadvantages of using PC-MRA for segmentation is that it is influenced by flow and its spatial resolution is relatively low. Alternatives are dedicated anatomical images [31]. A time-dependent measurement of the anatomy is usually dispensed with [31,32,33,34].

A combined one- and zero-dimensional ordinary differential equation (ODE) approach to model the entire Fontan circulation was recently calibrated and validated based on CMR and catheterisation data [35]. A one-dimensional fluid dynamics model based on 4D flow boundary conditions in the aorta suggested that patients with hypoplastic left heart syndrome have higher cerebral pressures and lower pressures and flow in the gut, with further differences between rest and exercise [36]. The most comprehensive approach to model haemodynamics is the detailed three-dimensional computational fluid dynamics (CFD) simulation, which models fluid dynamics details such as energy or power loss and blood flow distributions in order to identify adverse haemodynamic conditions [30,31,33,37].

A combination of 4D Flow MRI, CFD, and physical patient-specific models was used to assess the haemodynamics in TCPC anatomies [38]. Another CFD study validated by 4D flow demonstrated that aortopulmonary collateral flow significantly contributes to pulmonary artery flow distribution in patients with a Fontan circulation [39]. However, the validity of CFD depends strongly on boundary conditions, and a direct comparison with MRI may be affected by uncertainties [32].

### 2.4. Extended Reality

Extended reality (augmented reality, virtual reality, and mixed reality) is being increasingly used for procedural planning, teaching, and clinical assessment in patients with congenital heart disease [40,41]. The integration of 4D Flow data into an extended reality is possible and may offer further opportunities for blood flow assessment in Fontan patients. However, to our knowledge, few studies have been performed in non-Fontan patients so far [42]. We speculate that the main obstacle to displaying 4D flow using extended reality in clinical settings is the lack of suitable clinical software that supports spatio-temporal animation formats in addition to the two-dimensional video display. Beyond literature-based conclusions, we would like to name the available file formations for animations. STL and OBJ files cover only static geometries and the animation functionality of 3D-PDF is also limited, whereas 360° videos may include motion, but are limited by fixed perspectives, such that it is not possible to rotate or walk around the model. Possible formats that allow for the animation of 3D models from all perspectives are FBX, USD formats, or glTF/GL.B. The use of adequate animation formats may open future pathways for more interactive and immersive visualisations of flow data. We would like to encourage software manufacturers to provide suitable export functions.

## 3. Applications of 4D Flow in Fontan Patients

Four-dimensional Flow was used for a haemodynamic investigation of various aspects of the Fontan circulation.

### 3.1. Four-Dimensional Flow for Surgical and Interventional Planning

Four-dimensional Flow datasets are used for surgical and interventional planning, including for the Fontan patient group, although this is still limited to research studies and not yet widely clinically applied [39,43,44,45,46]. The first studies on this topic were published more than 20 years ago [47,48,49]. With the advancement of techniques, it is now possible to create patient-specific models and preoperatively model blood flow dynamics and blood flow distribution within the Fontan circulation [39,50].

### 3.2. Aortic Flow

**Figure 3:**
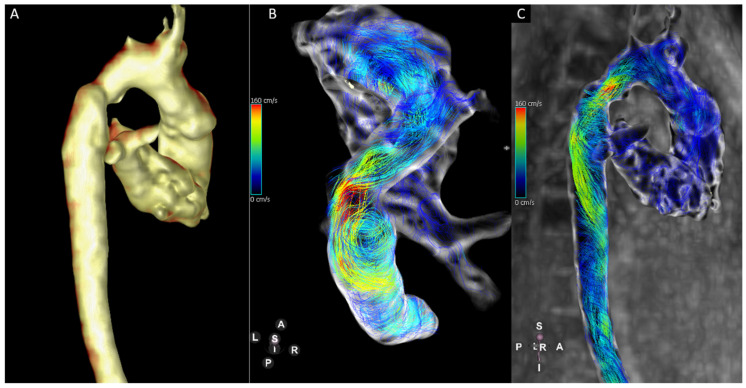
Thirteen-year-old patient with hypoplastic left heart syndrome. There are calibre discrepancies in the aortic arch (**A**). Four-dimensional Flow-derived stream lines show turbulent flow in the aortic arch with increased vortical flow in the mildly dilated descending aorta (**B**,**C**).

The neo-aorta was studied with respect to abnormal flow patterns and associated anatomy in several published analyses. It was found that sudden calibre changes in the section of the aortic arch of more than 40% were associated with extensive rotational flow in the entire thoracic descending aorta [51]. Rotational flow patterns in the descending aorta were further found to be associated with dilatation in the same section of the aorta (Figure 3).

Furthermore, a recent study found that gradual diameter changes are strongly linked to better clinical and haemodynamic outcomes compared to abrupt diameter changes [52].

Another study determined the curvature and twist of the neo-aorta in conjunction with fluid dynamics and noted an association between twist, abnormal flow patterns, and increased ventricular hypertrophy [53].

### 3.3. Ventricular Flow

Investigations on ventricular flow using 4D flow have been performed more recently in several disease entities, including Fontan patients. One of these studies determined the kinetic energy of the systemic ventricle in the cardiac cycle, finding that kinetic energy was influenced by the morphology of the ventricle and that diastolic kinetic energy indexed to stroke volume was decreased in Fontan patients compared to controls [54]. Decreased ventricular-kinetic-energy-altered intracardiac flow parameters were also found by others in mixed cohorts of Fontan patients [55,56]. Others measured intraventricular haemodynamic markers (kinetic energy, energy loss, and vorticity) using 4D Flow and dobutamine stress [57]. They found that that kinetic energy, energy loss, and vorticity increased during dobutamine stress and that there was a negative correlation with maximum oxygen uptake from cardiopulmonary exercise testing [57]. These findings underscore the dynamic nature of intracardiac flow, which may not be fully appreciated under resting conditions alone.

### 3.4. Total Cavopulmonary Connection (TCPC)

**Figure 4:**
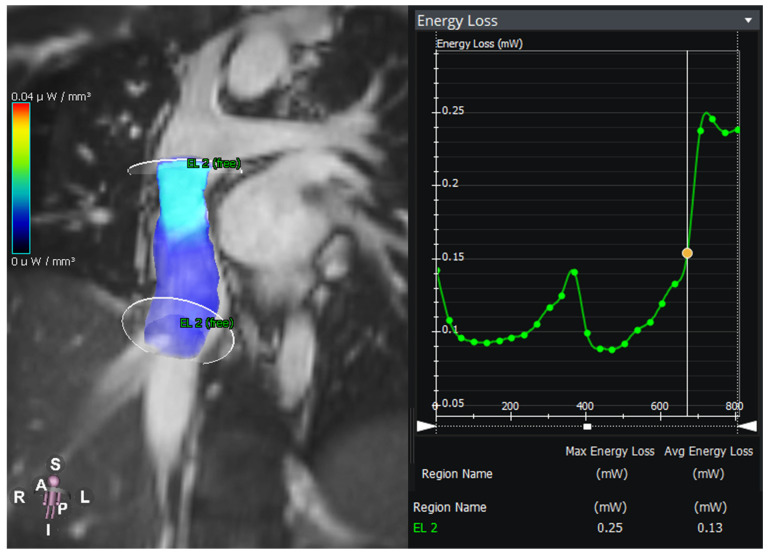
Energy loss in an intraatrial lateral tunnel in a patient with hypoplastic left heart syndrome. The graph shows the energy loss between the two planes over the time of the cardiac cycle.

The TCPC has been investigated under several aspects using 4D Flow (Figure 4). Blood flow measurements within the Fontan circuit and venovenous collateral flow can be reliably assessed [58]. Rijnberg et al. investigated vortical flow patterns and haemodynamic markers, including kinetic energy, viscous energy loss rate, and vorticity, at various levels of the TCPC [59]. They found that viscous energy loss rate was associated with vortical flow at the level of the LPA and Fontan confluence [59]. The same group showed in a recent study that kinetic energy and energy loss in the entire TCPC correlated positively with markers of liver fibrosis and negatively with peak oxygen consumption, suggesting that advanced 4D Flow markers might be clinically useful for the assessment of the efficiency of the Fontan circulation [60]. A potential source of increased energy loss within the TCPC is the confluence of the inferior vena cava, the hepatic veins, and the extracardiac conduit [61]. This may be related to adverse flow patterns in distorted geometry and inferior vena cava-to-conduit mismatch [61]. Others showed that an unequal flow distribution from the inferior vena cava to the pulmonary arteries may contribute to increased viscous energy loss in the caval veins, also suggesting that geometries play a role [62]. Inefficient blood flow might further be related to a blind pulmonary trunk [63].

The influence of respiration on the systemic venous return flow was comprehensively investigated and discussed in a CMR study by our group [17]. Furthermore, alternated TCPC haemodynamics have a strong influence on hepatic vein flow patterns, which may play an essential role in the hepatic pathophysiology of the Fontan circulation [17,64]. Recent studies using CMR flow acquisitions suggest that extracardiac conduits may be too small for adolescent and adult Fontan patients and that this may negatively impact the hepatic structure and might promote liver cirrhosis [65,66].

In the Fontan circulation, where systemic and pulmonary circulations are connected in series without a sub-pulmonary pump, the quantification and comparison of blood flow across different vascular compartments can yield clinically important insights. One key application is the detection and quantification of systemic-to-pulmonary collateral flow (SPCF), which represents blood that bypasses the heart and enters the pulmonary arteries from systemic sources. SPCF can be identified by comparing the pulmonary arterial inflow (typically measured at the branch pulmonary arteries) with the total pulmonary venous return (measured at the left and right pulmonary veins) [67]. Other measurement techniques have also been described [68]. A significant discrepancy indicates the presence of collateral vessels, which can contribute to volume overload and an impaired efficiency of the Fontan circuit [69]. Importantly, these flow measurements can be derived from 4D Flow CMR, allowing for comprehensive, time-resolved quantification within a single acquisition.

## 4. Discussion and Outlook

Four-dimensional flow is increasingly being used in patients with congenital heart disease, not only for research, but also for routine clinical assessment [70]. The technique is well suited for the evaluation of Fontan patients, as it allows for flow quantification, the visualisation of blood flow, 3D reconstructions, and the measurement of advanced haemodynamic parameters [71]. Thus, it can provide insights into Fontan physiology and pathophysiology not only in clinical research, but also in routine clinical CMR studies. This dual utility makes it an especially attractive tool in multidisciplinary care settings, where integrated data interpretation across cardiologists, radiologists, and surgeons can support more personalised treatment planning. Besides this, in conjunction with image-based simulation, 4D Flow can also be helpful in planning interventional and surgical procedures.

With all these advantages of 4D Flow, there are also some disadvantages. These include the relatively long scan times with conventional sequences and the need for 4D Flow post-processing software. In Fontan patients, typically, different flow velocities in the TCPC and the aorta must also be taken into account, as without the availability of multi- VENC sequences, this requires the acquisition of separate 4D flow data sets.

However, 4D Flow has proven its clinical utility, at least for standard blood flow assessments. Future work should be performed to implement advanced haemodynamic markers into the routine clinical practice. As 4D Flow MRI continues to evolve, its role in clinical practice is expected to expand, with ongoing advancements in hardware and software allowing for faster, more accurate assessments. Modelling studies using 4D Flow and computational fluid dynamics might improve the prediction of Fontan haemodynamics and outcomes. Machine learning algorithms are expected to play a significant role in automating image processing and flow quantification, reducing the time required for post-processing and enhancing the accuracy of measurements [72,73]. Furthermore, the integration of machine learning into image-based simulations and CFD modelling could improve the predictive power of 4D Flow in assessing long-term outcomes in Fontan patients.

## 5. Limitations

The limitations of the current study include potential selection bias in the included studies, as well as incomplete coverage of the available literature. No standardised assessment of the quality of the referenced studies was performed. Furthermore, the review may be biased by the authors’ views and perspectives.

## 6. Conclusions

Four-dimensional Flow MRI is a method that is becoming increasingly popular. It can be easily acquired and is useful for assessing advanced haemodynamics in patients with a Fontan circulation, both for clinical assessments and for research purposes. This review provides an overview of its various applications and considers future directions. Further work is needed to implement 4D Flow research methodologies in clinical practice.

## Data Availability

No new data were created or analysed in this study. Data sharing is not applicable to this article.

## Abbreviations

The following abbreviations are used in this manuscript:

4D	Four-dimensional
TCPC	Total cavopulmonary connection
3D	Three-dimensional
2D	Two-dimensional
VENC	Velocity encoding
